# Functional Analysis of 3-Dehydroquinate Dehydratase/Shikimate Dehydrogenases Involved in Shikimate Pathway in *Camellia sinensis*

**DOI:** 10.3389/fpls.2019.01268

**Published:** 2019-10-11

**Authors:** Keyi Huang, Ming Li, Yajun Liu, Mengqing Zhu, Guifu Zhao, Yihui Zhou, Lingjie Zhang, Yingling Wu, Xinlong Dai, Tao Xia, Liping Gao

**Affiliations:** ^1^School of Life Science, Anhui Agricultural University, Hefei, China; ^2^State Key Laboratory of Tea Plant Biology and Utilization, Anhui Agricultural University, Hefei, China

**Keywords:** *Camellia sinensis*, 3-dehydroquinate dehydratase/shikimate dehydrogenase, gallic acid, shikimate pathway, site-directed mutagenesis

## Abstract

Polyphenols play an important role in the astringent taste of tea [*Camellia sinensis* (L.)] infusions; catechins in phenolic compounds are beneficial to health. The biosynthesis of gallic acid (GA), a precursor for polyphenol synthesis, in tea plants remains unknown. It is well known that 3-dehydroquinate dehydratase/shikimate dehydrogenase (DQD/SDH) is a key enzyme for catalyzing the conversion of 3-dehydroshikimate (3-DHS) to shikimate (SA); it also potentially participates in GA synthesis in a branch of the SA pathway. In this study, four *Cs*DQD/SDH proteins were produced in *Escherichia coli*. Three *Cs*DQD/SDHs had 3-DHS reduction and SA oxidation functions. Notably, three *Cs*DQD/SDHs showed individual differences between the catalytic efficiency of 3-DHS reduction and SA oxidation; *Cs*DQD/SDHa had higher catalytic efficiency for 3-DHS reduction than for SA oxidation, *Cs*DQD/SDHd showed the opposite tendency, and *Cs*DQD/SDHc had almost equal catalytic efficiency for 3-DHS reduction and SA oxidation. *In vitro*, GA was mainly generated from 3-DHS through nonenzymatic conversion. Quantitative reverse transcriptase polymerase chain reaction (qRT-PCR) analysis showed that *CsDQD/SDHc* and *CsDQD/SDHd* expression was correlated with GA and 1-*O*-galloyl-β-D-glucose accumulation in *C. sinensis*. These results revealed the *Cs*DQD/SDHc and *Cs*DQD/SDHd genes are involved in GA synthesis. Finally, site-directed mutagenesis exhibited the mutation of residues Ser-338 and NRT to Gly and DI/LD in the SDH unit is the reason for the low activity of *Cs*DQD/SDHb for 3-DHS reduction and SA oxidation.

## Introduction

**Figure 1:**
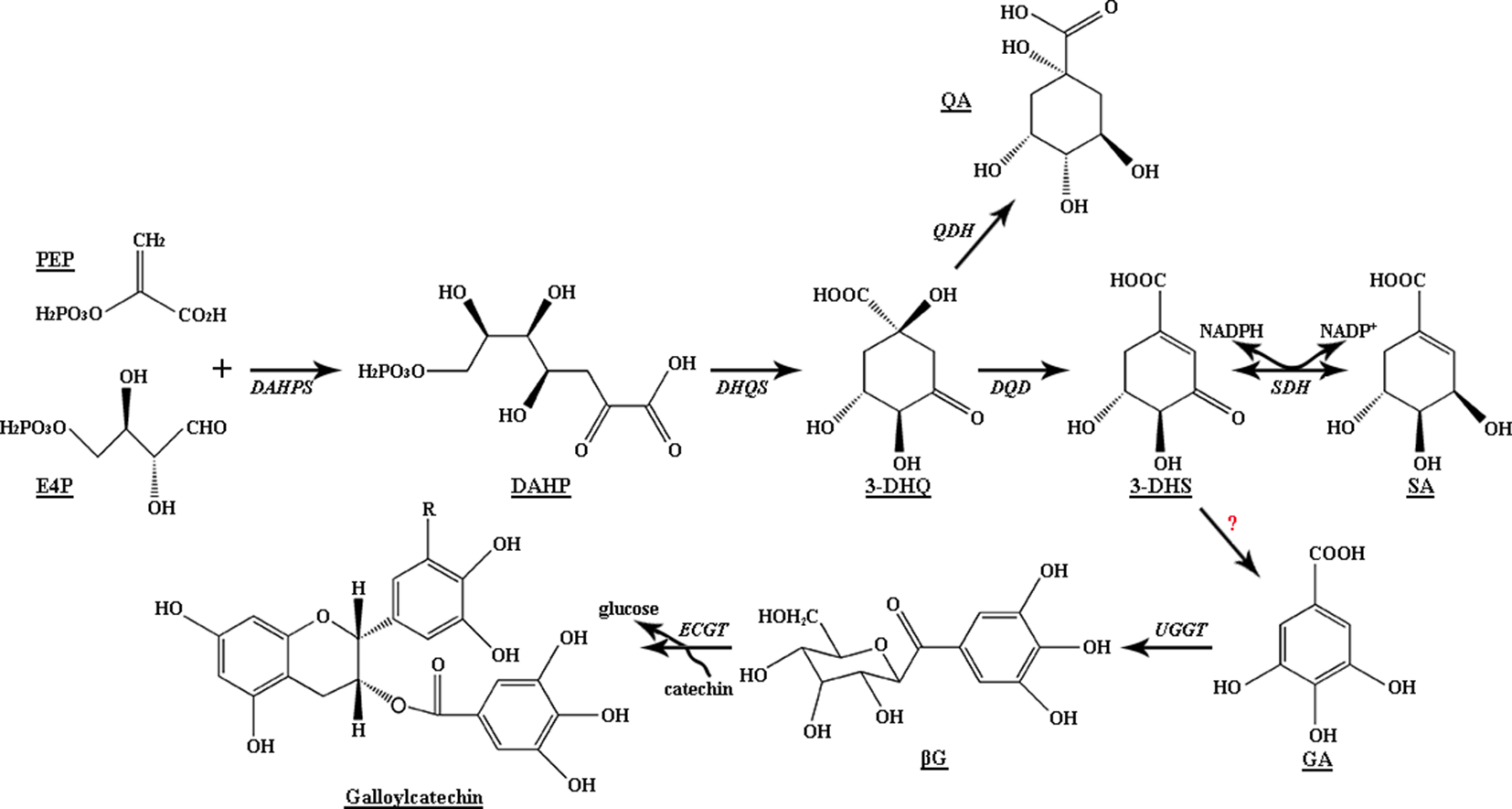
Biosynthetic pathway of gallic acid and shikimic acid in plants. DAHPS, 3-deoxy-D-arabino-heptulosonate 7-phosphate synthase; DHQS, 3-dehydroquinate synthase; DQD, 3-dehydroquinate dehydratase; SDH, SA dehydrogenase; QDH, quinate dehydrogenase; UGGT, UDP-glucose: gallate 1-*O*-galloyltransferase; ECGT, epicatechin: 1-*O*-galloyl-β-D-glucose *O*-galloyltransferase.

The shikimate (SA) pathway contributes to the production of a wide range of intermediates and aromatic amino acids vital for secondary metabolites and protein biosynthesis of microorganisms and plants. The SA pathway in plants consists of seven enzymatic reaction steps, beginning with the condensation of phosphoenolpyruvate (PEP) and erythrose 4-phosphate to form 3-deoxy-d-arabino-heptulosonate 7-phosphate (DAHP) and ending with the synthesis of chorismate from 5-enolpyruvylshikimate 3-phosphate (EPSP) (**Figure 1**) (Sprenger, 2006; Bentley and Haslam, 2008). Chorismic acid, the end-product of SA pathway, is essential for the formation of aromatic amino acids (l-tyrosine, l-tryptophan, and l-phenylalanine), which can be further catalyzed into several secondary metabolites, such as phenylalanine, lignin, flavonoids, chlorogenic acid, indole acetic acid, and alkaloids (Sprenger, 2006; Maeda and Dudareva, 2012). Tyrosine and phenylalanine are two crucial precursors that can be catalyzed by phenylalanine ammonia-lyase (PAL) to activate the phenylalanine pathway and accumulate flavonoids, polyphenols, and lignin. Furthermore, increasing *FaSKDH* expression can activate the phenylpropanoid pathway to promote flavonoid and polyphenol accumulation in strawberry fruit (Xu et al., 2014; Nagpala et al., 2016).

The SA pathway is important to plant growth, development, and defense; and silencing the DAHP synthase gene (*DAHPS*) or 3-dehydroquinate dehydratase/shikimate dehydrogenase (*DQD/SDH*) in potato, tomato, and tobacco plants results in slow growth and reduces the production of secondary metabolites, such as lignin and chlorogenic acid (Dyer et al., 1989; Kasama et al., 2007). In plants, DQD/SDH, a bifunctional enzyme, is crucial in the third and fourth reversible reactions in the SA pathway; SA can be formed from 3-dehydroshikimate (3-DHS) and NADPH under catalysis by DQD/SDH, and 3-DHS can also be generated from SA and NADP^+^ under catalysis by DQD/SDH (Kasama et al., 2007; Maeda and Dudareva, 2012). Analysis of the crystal structure of *Arabidopsis* DQD/SDH showed the functional difference between DQD and SDH sites, which bind with the substrates SA and tartrate to form complexes (Singh, 2006), respectively. Metabolic flux through the SA pathway can be controlled by increasing the effective concentration of the intermediate 3-DHS at each of the two sites. The ratio of SDH to DQD catalytic efficiency is 9:1; therefore, 3-DHS can be quickly converted to SA without accumulation in the stroma of spinach chloroplasts (Fiedler and Schultz, 1985).

Polyphenols in tea plants, including phenolic acids, catechins, and flavonol derivatives, not only determine the mouthfeel of tea infusions but also provide health benefits. Tea plants have abundant ester catechin and epigallocatechin gallate (EGCG) content, which range up to 12%. Gallic acid (GA) is an essential precursor for galloylated catechin biosynthesis (**Figure 1**), and the amount of GA is a key limiting factor for the formation of EGCG and epicatechin gallate (ECG) (Liu et al., 2012). In addition, GA derivatives, including hydrolysable tannins (HTs) and mainly galloylquinic acid (GQA), highly accumulate in vegetables, *Rhus typhina*, and *Camellia sinensis* (Haslam and Cai, 1994; Niemetz and Gross, 2005; Jiang et al., 2013). They are responsible for the unique flavor of plant-derived foods and show a wide range of biological activities, including antioxidant, antiviral, anti-inflammatory, antibacterial, anticancer, and immune-regulation activities (Vit et al., 2008; Steinmann et al., 2013).

GA biosynthesis has been studied for more than 50 years since the 1960s (Dewick and Haslam, 1969). By using the isotope-labeling method, studies have provided an indication regarding GA biosynthesis: GA is mainly derived from the dehydrogenation of 3-DHS by the action of SDH to produce 3,5-didehydroshikimate. This compound tautomerizes to form the redox equivalent GA. Studies have also verified this speculation in plants: GA could be synthesized from 3-DHS with NADP^+^ as the cofactor when the crude enzyme of birch leaf was used (Ossipov et al., 2003). Transgenic *Nicotiana tabacum* lines overexpressing *Juglans regia* *SDH* exhibited a 500% increase in GA accumulation (Muir et al., 2011). In the persimmon fruits of pollination-constant and nonastringent (PCNA)-type mutants, downregulated *SDH* expression was correlated with the reduction in epigallocatechin content, which confirmed the correlation of SDH with proanthocyanidin (PA) content (Akagi et al., 2009). Recently, two of the four *Vv*SDH genes, namely, *Vv*SDH3 and *Vv*SDH4, were validated to be involved in GA biosynthesis in Vitis vinifera (Bontpart et al., 2016).

Recently, an early intermediate of the SA pathway, 3-DHS, was identified to be a crucial precursor for GA synthesis in plants. Alternatively, a study found that GA could be produced through the SA pathway and phenylpropanoid pathway simultaneously. Ishikura and coworkers revealed that GA was derived from not only labeled SA in young leaves but also labeled l-phenylalanine in mature and autumn leaves of *Acer buergerianum* and *Rhus succedanea* (Ishikura et al., 1984); this finding indicated that the biosynthetic pathway of GA is diverse and changes according to the developmental stage of the plant.

Studies related to the key gene involved in GA biosynthesis in tea plants are scant. In this study, we screened out four *CsDQD/SDH* genes from the tea genome (designated as *CsDQD/SDHa*, *CsDQD/SDHb*, *CsDQD/SDHc*, and *CsDQD/SDHd*). The functions of *CsDQD/SDHs* expressed in *Escherichia coli* were surveyed, and after purification, the capacity of recombinant proteins *in vitro* to produce GA was determined using enzymatic analysis. We also evaluated the relationship between the expression patterns of these four *CsDQD/SDH* genes and the contents of GA and its derivatives in various organs of tea plants. The results may provide a basis for understanding the biosynthesis of GA and its derivatives.

## Methods and Materials

### Plant Material

*Camellia sinensis* cv. Shuchazao (variety approval number: CHN20022008) samples were obtained from the experimental tea garden of Anhui Agricultural University, Hefei, China. Leaves (bud, first leaf, and second leaf), young stems, and tender roots were immediately frozen in liquid nitrogen and stored at −80°C until use.

### Chemicals

DL-Dithiothreitol (DTT), ethylenediaminetetraacetic acid (EDTA), NADP^+^, NADPH, GA, SA, 3-DHS, NaCl, NaOH, Tris, maltose, HCl, isopropyl β-D-thiogalactoside (IPTG), Bis-Tris propane HCl (BTP-HCl), tryphone, and yeast extract were obtained from Sigma Aldrich (St. Louis, MO, USA). Ultra-performance liquid chromatography (UPLC)-grade methanol, acetic acid, acetonitrile, and phosphoric acid were purchased from Tedia Co., Ltd. (Fairfield, OH, USA).

### *CsDQD*/*SDH* Cloning and Sequence Analysis

Total RNA was isolated from 50 mg of various tea tissues using the RNAiso Plus kit and RNAiso-mate (Takara, Dalian, China), and cDNA was synthesized using the PrimeScript RT Reagent Kit (Takara, Dalian, China) according to the manufacturer’s protocol. The sequences of *CsDQD/SDHa* (National Center for Biotechnology Information (NCBI) accession number: MH000201, 1,605 bp), *CsDQD/SDHb* (NCBI accession number: MH000202, 1,560 bp), and *CsDQD/SDHc* (NCBI accession number: MH000203, 1,599 bp) were obtained from the NCBI database, and the sequence of *CsDQD/SDHd* (NCBI accession number: MH000204, 1,578 bp) was obtained from the Sequence Read Archive database at NCBI under Bioproject ID PRJNA283013 for Huangjinya. The open reading frames (ORFs) of *CsDQD/SDHs* were cloned with high-fidelity DNA polymerase using *C. sinensis* cv. Shuchazao cDNA as the template and were constructed into a pEASY-Blunt Simple Cloning Kit vector (New England Biolabs, MA, USA) to sequence the full-length gene. The forward and reverse primers for cloning into the pMAL-c2x expression vector (New England Biolabs) were inserted to incorporate the restriction site for *Bam*HI and *Pst*I before the start codon and after the stop codon, respectively (primers are listed in **Supplementary Table S1**).

Multiple DQD/SDH protein sequences (**Supplementary Table S2**) were obtained from Phytozome (https://phytozome.jgi.doe.gov/pz/portal.html) 12.0 and NCBI (https://www.ncbi.nlm.nih.gov/). Amino acid sequences were aligned, and phylogenetic analysis was performed using DNAMAN 6.0 (Lynnon Corporation, San Ramon, CA, USA), Primer 5.0 (Primer, Canada), and MEGA 5.0 (Mega, Raynham, MA, USA) (Tamura et al., 2011). The tree nodes were statistically evaluated using the bootstrap method, with 1,000 bootstrap replicates conducted. A neighbor-joining tree with the evolutionary distance was computed using the ρ-distance model and protein sequences alignment with a gap open penalty of 10 and a gap extension penalty of 0.2.

### *CsDQD*/SDH Expression in *Escherichia coli* and Purification of Recombinant Proteins

*CsDQD/SDH* ORFs were cloned into the pMAL-c2x (New England Biolabs, MA, USA) expression vector and then expressed in *E. coli* NovaBlue (DE3) competent cells (Novagen, Schwalbach, Germany) to express recombinant proteins, and the proteins purified according to the manufacturer’s protocol (New England Biolabs). The recombinant *E. coli* strains expressing *Cs*DQD/SDHs were shaken in Luria–Bertani (LB) medium (1 L of LB culture medium containing 5 g of yeast extract, 10 g of tryptone, and 10 g of NaCl, adjusted to pH 7.0 before sterilization) containing 1 µL·ml^−1^ of ampicillin to an OD_600_ of 0.6, and the cells were shaken at 37°C at 220 rpm. Thereafter, 1 mmol·L^−1^ of IPTG was added to the culture medium, and protein expression in strains was induced at 24°C for 24 h. After centrifugation at 7,200 rpm for 15 min, supernatants were discarded, and the precipitates were resuspended in column buffer (200 mM of NaCl, 20 mM of Tris, 1 mM of EDTA, and 1 mM of DTT, pH 7.5). The cells were ultrasonically disrupted for approximately 20 min and then centrifuged at 6,500 rpm for approximately 15 min at 4°C (SCIENTZ-llD, NingBo, China). Supernatants were purified using a maltose column; finally, the target proteins were eluted using maltose column buffer (20 mM of maltose in column buffer, pH 7.5). The target proteins were concentrated using 50-kD ultrafiltration concentration pipes. Recombinant protein concentrations were determined using Coomassie Brilliant Blue G-250, and their concentrations were confirmed by running the proteins on sodium dodecyl sulfate–polyacrylamide gel electrophoresis (SDS-PAGE) electrophoresis gel; they were then stored at −80°C in 50% glycerol.

### Enzyme Activity and Product Analysis

To analyze the *in vitro* activity of the candidate *Cs*DQD/SDHs for 3-DHS reduction and SA oxidation, reactions were conducted in a 100-µL reaction solution consisting of 100 mM of BTP-HCl buffer (pH 7.5), 1 mM of NADPH or NADP^+^ as the cofactor donor, 1 mM of 3-DHS or 1 mM of SA as the substrate, and 10 µg of purified recombinant *Cs*DQD/SDHs protein at 30°C for 30 min. Reactions were stopped by mixing the reaction solutions with 2.3 M of HCl. Reaction samples lacking recombinant proteins were used as blank controls.

The high-performance liquid chromatography (HPLC) system from Agilent Technologies (RHMo Alto, CA, USA) was used in this study to detect 3-DHS and SA (UV maximum absorption wavelengths were 234 and 211 nm, respectively). The HPLC system comprised a Venusil XBP C18 reverse phase column (4.6 × 251 mm^2^, Agela Technologies), quaternary pump with a vacuum degasser, thermostated column compartment, and autosampler. The elution profile was as follows: 100% eluent A (1% phosphoric acid in water) for 0–24 min and termination at 25 min at 0.2 mL·min^−1^ flow rate (eluent B: 100% acetonitrile).

To analyze the *in vitro* activity of *Cs*DQD/SDHs to produce GA, assays were performed in a buffer containing 100 mM of BTP-HCl buffer (pH 9.0), 1 mM of 3-DHS, 1 mM of NADP^+^, and 10 µg of purified recombinant *Cs*DQD/SDHs protein at 30°C for 30 min. Reactions were stopped by mixing the reaction solutions with an equal volume of 100% methanol. GA (UV maximum absorption wavelengths were 280 nm) was analyzed using a reverse-phase HPLC LC10Avp system (Shimadzu, Kyoto, Japan). The column was eluted using a mobile phase consisting of eluent A (1% acetic acid) and eluent B (100% acetonitrile) at room temperature. The elution profile was as follows: starting with 100% A (1% acetic acid), a linear gradient from 1% to 5% B (100% acetonitrile) for 0–8 min, 5–10% B for 8–13 min, 10–1% B for 13–14 min, and termination at 15 min at a flow rate of 0.2 ml·min^−1^ (eluent B: 100% acetonitrile).

All products were analyzed using UPLC–triple quadrupole mass spectrometry (QQQ)–tandem mass spectrometry (MS/MS) with an Agilent 20RBAX RRHD Eclipse Plus C18 column (particle size, 1.8 mm; length, 100 mm; and internal diameter, 2.1 mm) at a flow rate of 0.4 ml·min^−1^ following previously published protocols (Jiang et al., 2013; Zhao et al., 2017). All reactions in each experiment were three bio-replications.

### Enzymatic Kinetic Analysis of Recombinant *CsDQD*/SDH Proteins

Optimal pH analysis was performed using 3-DHS and SA as substrates at varying pH. The buffers for the pH test were 100 mM of citric acid with pH ranging from 4 to 7, 100 mM of BTP-HCl buffer with pH ranging from 6 to 9, and 100 mM of sodium carbonate with pH ranging from 8 to 11. The reaction was maintained at 30°C for 30 min, and the reaction was stopped by adding 40 µL of 2.3 M of HCl.

*In vitro*, the kinetic parameters of the recombinant enzymes were obtained from hyperbolic Michaelis–Menten saturation curves for substrates. For the measurement of the *K*_M_ and *V*_max_ of *Cs*DQD/SDHs, 3-DHS, SA, NADP^+^, and NADPH were used as acceptor substrates. The linear phase of the reaction was conducted in BTP-HCl buffer (pH 8.5) with 1.5 mM of 3-DHS and NADP^+^ (0–500 µM), 1.5 mM of NADP^+^ and 3-DHS (0–500 µM), 1.5 mM of SA and NADPH (0–500 µM), and 1.5 mM of NADPH and SA (0–500 µM) at 30°C for 3 min. Three biological replicates were used per reaction.

### Functional Unit Reorganization and Site-Directed Mutagenesis

To identify the key unit and amino acid residues responsible for 3-DHS reduction and SA oxidation, *Cs*DQD/SDHa was categorized as two mutant proteins named *Cs*DQDa (from Lys-91 to Phe-316 in the *At*SDH protein sequence) and *Cs*SDHa (from IIe-328 to Gly-588 in the *At*SDH protein sequence). Referring to the model of *Arabidopsis thaliana* protein crystal structure, the amino acid residue sites of Gly-338, Gly-381, Asp-483, Leu-484, and Asp-485 in *Cs*DQD/SDHb were mutated to Ser-338, Thr-381, Asn-483, Arg-484, and Thr-485, respectively; the mutant protein was named MT*Cs*DQD/SDHb.

Site-directed mutagenesis was performed using a gene site-directed mutagenesis kit. The plasmid pMAL-c2X harboring *Cs*DQD/SDHs was used as templates to obtain the site-directed mutants of *Cs*DQDa, *Cs*SDHa, and MT*Cs*DQD/SDHb. Oligonucleotide sequences specifically designed for mutagenesis are listed in **Supplementary Table S1**.

Purification and analysis of the mutant recombinant proteins were performed using the same protocols as those used for native proteins. The quantitative measurement of the recombinant enzyme products was performed using the aforementioned method, and three biological replicates were used for each experiment.

### Expression of GA and GA-Related Compounds and Their Accumulation in Tea Plants

GA, βG, ECG, and EGCG were extracted from various organs of tea plants as follows: 1 g of the dry weight sample (bud, first leaf, second leaf, stem, and root) was ground in liquid nitrogen; it was then extracted with 2 ml of the extraction solution (0.2% HCl added to 80% methanol and 20% water) at room temperature through ultrasonic extraction for 10 min and centrifugation for 15 min at 4,000 rpm (Wang et al., 2018). The precipitate was resuspended in the extraction solution and re-extracted twice as previously described; supernatants were filtered through a 0.22-µm filter membrane. Three biological replicates were analyzed using quantitative reverse transcriptase polymerase chain reaction (qRT-PCR).

GA, βG, ECG, and EGCG contents were detected using an UPLC–MS/MS system equipped with a quaternary pump with a vacuum degasser, thermostated column compartment, autosampler, diode array detector (DAD), and QQQ purchased from Agilent Technologies (Palo Alto, CA, USA). Samples were analyzed using UPLC–QQQ–MS/MS with an Agilent 20RBAX RRHD Eclipse Plus C18 column (particle size, 1.8 mm; length, 100 mm; and internal diameter, 2.1 mm) at a flow rate of 0.4 mL·min^−1^ following previously published protocols (Jiang et al., 2013; Zhao et al., 2017).

### Expression Pattern of *CsDQD/SDHs* in Tea Plants

The expression levels of glyceraldehyde-3-phosphate dehydrogenase gene (*GAPDH*), as the reference gene, were standardized against *CsDQD/SDHs* expression levels. Total RNA was extracted using TRIzol and then reverse transcribed into cDNA. All primers were designed using Primer 5.0 and were detected using PCR. The qRT-PCR system contained 200 ng of cDNA template, 10 µL of IQ SYBR Green Supermix (Takara), and 0.8 µL of each gene-specific primer, and the reaction volume of 20 µL was attained through the addition of RNase-free H_2_O. The expression levels are represented as a mean value of three replicates. Relative *CsDQD/SDH* expression was deduced from the cycle threshold (CT) based on the 2^−ΔΔCt^ method. ΔCT = CT_target_ − CT_internal standard_ and −ΔΔCT = −(ΔCT_target_ − ΔCT_control_), where CT_target_ and CT_internal standard_ are the cycle threshold (CT) values for the target and housekeeping genes, respectively. The Pearson correlation coefficient of the *CsDQD/SDH* expression profiles was also calculated.

## Results

### Cloning and Protein Sequence Analysis of Four *CsDQD/SDH* Genes

The four *CsDQD/SDH* genes were screened from the NCBI and tea genome database and successfully cloned from the tea plant cDNA library. Their ORF lengths are 1,605, 1,560, 1,599, and 1,578 nucleotides, and their encoded proteins are 534, 519, 532, and 525 amino acid residues in length, respectively, with predicted molecular weights of 57.568, 56.411, 58.241, and 56.776 kDa, respectively, and calculated isoelectric points (pIs) of 6.26, 6.1, 6.73, and 6.8, respectively.

Analysis using DNAMAN 6.0 showed that the protein sequence of DQD/SDHs in plants shared 54.17% consistency (**Supplementary Figure 1**). The amino acid sequence of *Cs*DQD/SDHa shared 64.11% and 66.79% identity with *Cs*DQD/SDHc and *Cs*DQD/SDHd. However, *Cs*DQD/SDHb showed only 49.44%, 48.41%, and 48.67% identity with *Cs*DQD/SDHa, *Cs*DQD/SDHc, and *Cs*DQD/SDHd, respectively.

**Figure 2:**
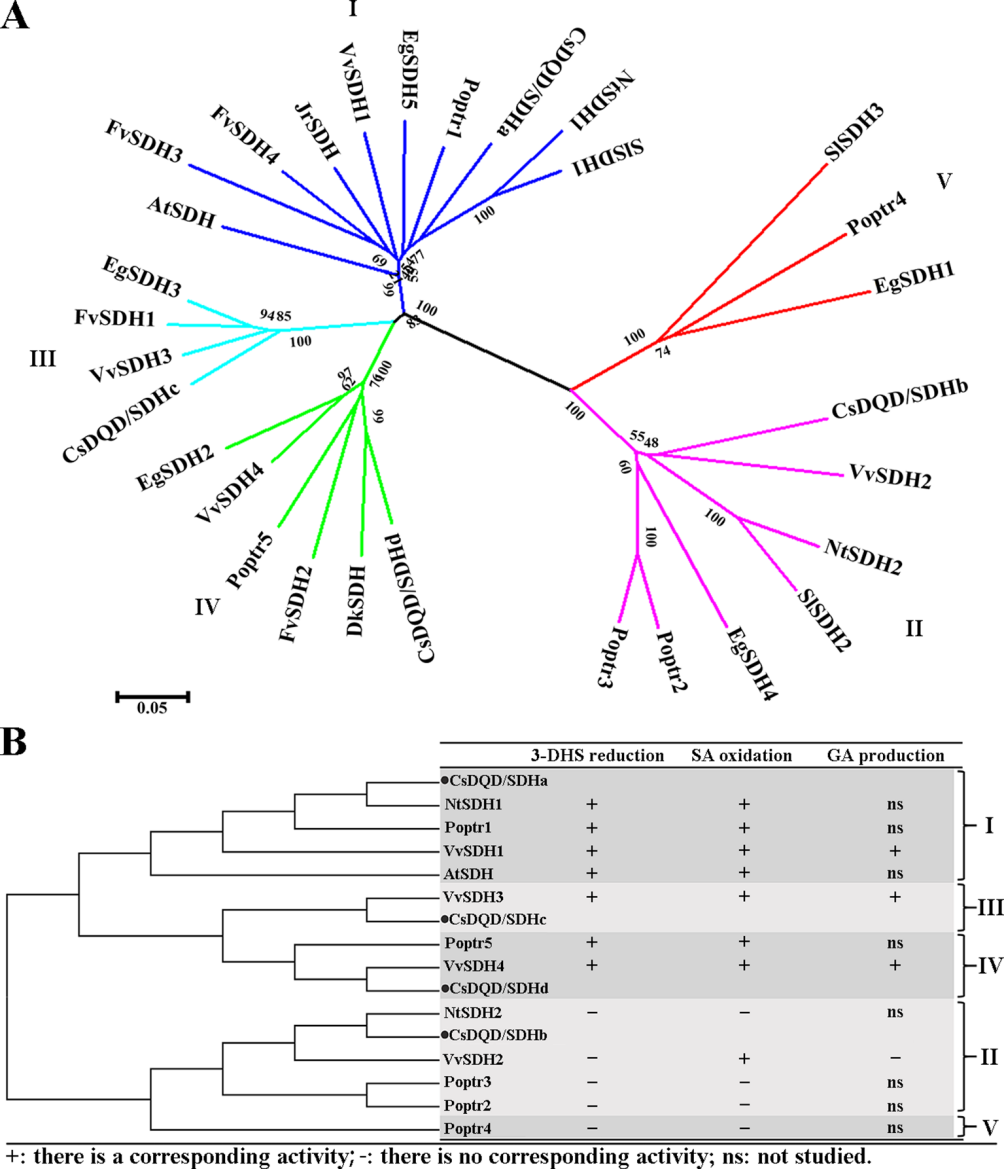
Neighbor-joining tree analysis. **(A)** A neighbor-joining tree was constructed from the four *Cs*DQD/SDHs sequenced in this study and the 26 sequences available in public databases (NCBI and Phytozome 12) using MEGA 5.0 software. **(B)** A simplified neighbor-joining tree was constructed to display the enzyme characteristics of each DQD/SDH protein from *Arabidopsis thaliana*, *Nicotiana tabacum*, *Vitis vinifera*, and *Populus trichocarpa*. NCBI, National Center for Biotechnology Information; DQD/SDH, 3-dehydroquinate dehydratase/shikimate dehydrogenase.

Some characterized DQD/SDH protein sequences were extracted from the NCBI database and Phytozome 12.0, and a phylogenetic tree was constructed using MEGA 5.0 (**Figure 2A**).The result showed that the plant DQD/SDH proteins can be divided into five groups. Bontpart et al. (2016) divided *Vv*SDH proteins into four groups. The *Cs*DQD/SDH proteins in this study were divided into four groups, similar to *Vv*SDH proteins. **Figure 2B** presents the enzyme characteristics of each DQD/SDH protein identified from *Arabidopsis thaliana* (Singh, 2006), *Nicotiana tabacum* (Kasama et al., 2007), *Vitis vinifera* (Bontpart et al., 2016), and *Populus trichocarpa* (Guo et al., 2014). Relevant studies have indicated that the DQD/SDH proteins in the first, third, and fourth groups derived from showed both 3-DHS reduction and SA oxidation activities. However, the enzymatic activities of several DQD/SDH proteins in the second group could not been detected. *Vv*SDH2 only showed a very low SA oxidation activity (Bontpart et al., 2016). Whether DQD/SDH proteins participate in GA production needs further investigation and supporting evidence, although research has proved that *Vv*SDH3 and *Vv*SDH4 are involved in GA production *in vitro* (Bontpart et al., 2016).

The DQD/SDH protein sequence alignments in **Figure 2** show that the plant DQD/SDH proteins consist of two functional units: the DQD unit (**Supplementary Figure 2A**) and the SDH unit. The DQD unit can catalyze the conversion of 3-dehydroquinate to 3-DHS (Michel et al., 2003; Vogan, 2003), whereas the SDH unit can catalyze the NADPH-dependent reduction of 3-DHS to SA (Padyana and Burley, 2003; Ye et al., 2003). According to the structure of *At*DQD/SDH (Singh, 2006), residues Lys-241 and His-214 as well as Arg-279 in the DQD unit function as key catalytic groups and a binding group, respectively. Residues Lys-385 and Asp-423 have been proposed to be a catalytic dyad in the SDH unit, and Ser-336 has been proposed to be its key binding group. Moreover, Asn-483, Arg-484, and Thr-485 formed the NRT motif responsible for binding the cofactor NADP(H). The Ser-338 Ala mutant of *At*SDH affected substrate binding and its catalysis. These amino acid residues were conserved in plant DQD/SDHs of the first, third, and fourth groups, except for *Cs*DQD/SDHc (where His-214 was replaced by Gln).

Notably, the corresponding catalytic residues His-214 and Arg-279 in the DQD unit and Ser-338 in the SDH unit of *Cs*DQD/SDHb and *Vv*SDH2 belonging to the second group were replaced by Tyr, Gln, and Gly, respectively. The position of NRT was replaced by DI/LD in *Cs*DQD/SDHb and *Vv*SDH2. These results may contribute to the difficulty in detecting the enzymatic activities of DQD/SDH proteins in the second group.

### Enzyme Assays and Product Identification

**Figure 3:**
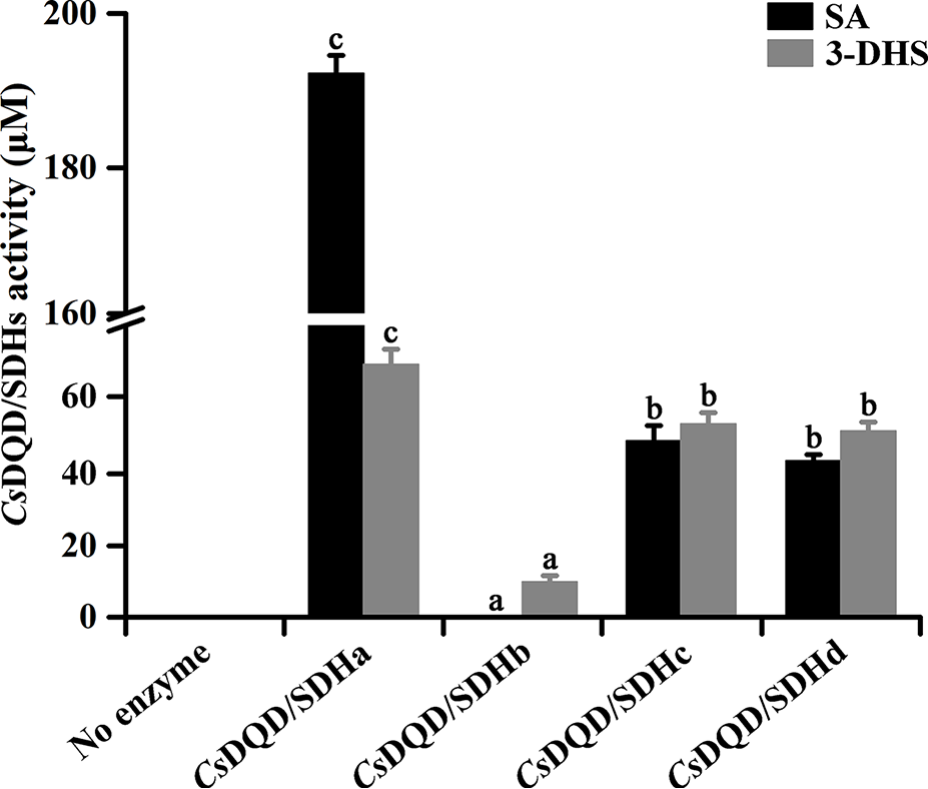
Quantification of 3-DHS and SA production catalyzed by recombinant *Cs*DQD/SDHs *in vitro*. The reaction products in enzymatic assays were quantified using HPLC (*λ*_3-DHS_ = 234 nm, *λ*_SA_ = 211 nm). Data are shown as the mean of three replicates ± SD. Different letters represent statistically differ at *P* < 0.05 according to one-way ANOVA of Duncan’s test. 3-DHS, 3-dehydroshikimate; SA, shikimate; HPLC, high-performance liquid chromatography; ANOVA, analysis of variance.

To detect a recombinant protein activity, the four *Cs*DQD/SDHs were fused to maltose-binding protein and expressed in NovaBlue (DE3) strains. The NADPH-dependent reduction of 3-DHS (**Supplementary Figure 3A**) and the NADP^+^-dependent oxidation of SA (**Supplementary Figure 3B**) by the recombinant proteins of *Cs*DQD/SDHa were measured, and the corresponding products in these reactions were detected quantitatively through UPLC–QQQ–MS/MS (**Supplementary Figure 3C**). Quantitative results indicated that among these four recombinant proteins, *Cs*DQD/SDHa exhibited the highest 3-DHS reduction and SA oxidation activities, and *Cs*DQD/SDHb exhibited the lowest SA oxidation activity but had no 3-DHS reduction activity (**Figure 3**).

**Figure 4:**
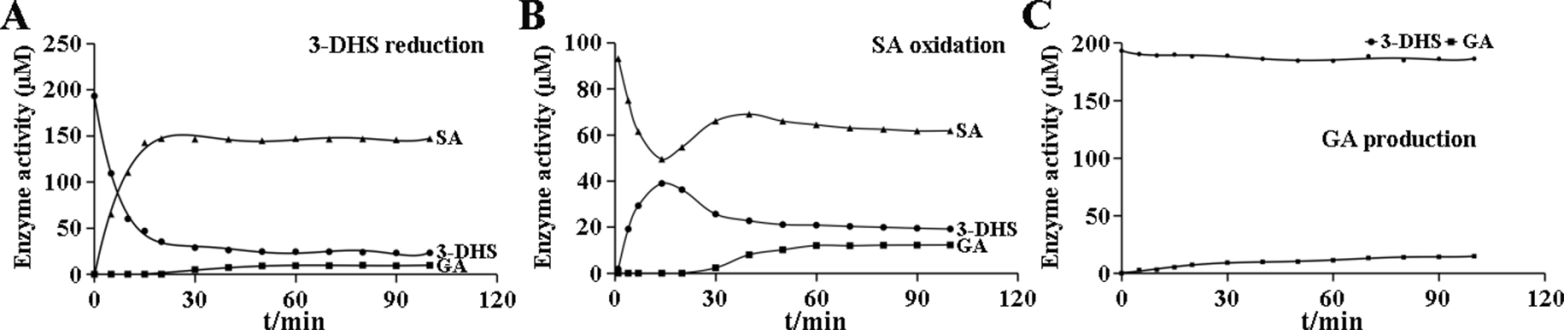
Enzymatic reaction time of the recombinant protein *Cs*DQD/SDHa *in vitro*. **(A)** The time course of the 3-DHS reduction reaction catalyzed by the *Cs*DQD/SDHa enzyme. **(B)** The time course of the SA oxidation catalyzed by the *Cs*DQD/SDHa enzyme. **(C)** GA production in a reaction catalyzed by the *Cs*DQD/SDHa enzyme. 3-DHS, 3-dehydroshikimate; SA, shikimate; GA, gallic acid.

**Figure 5:**
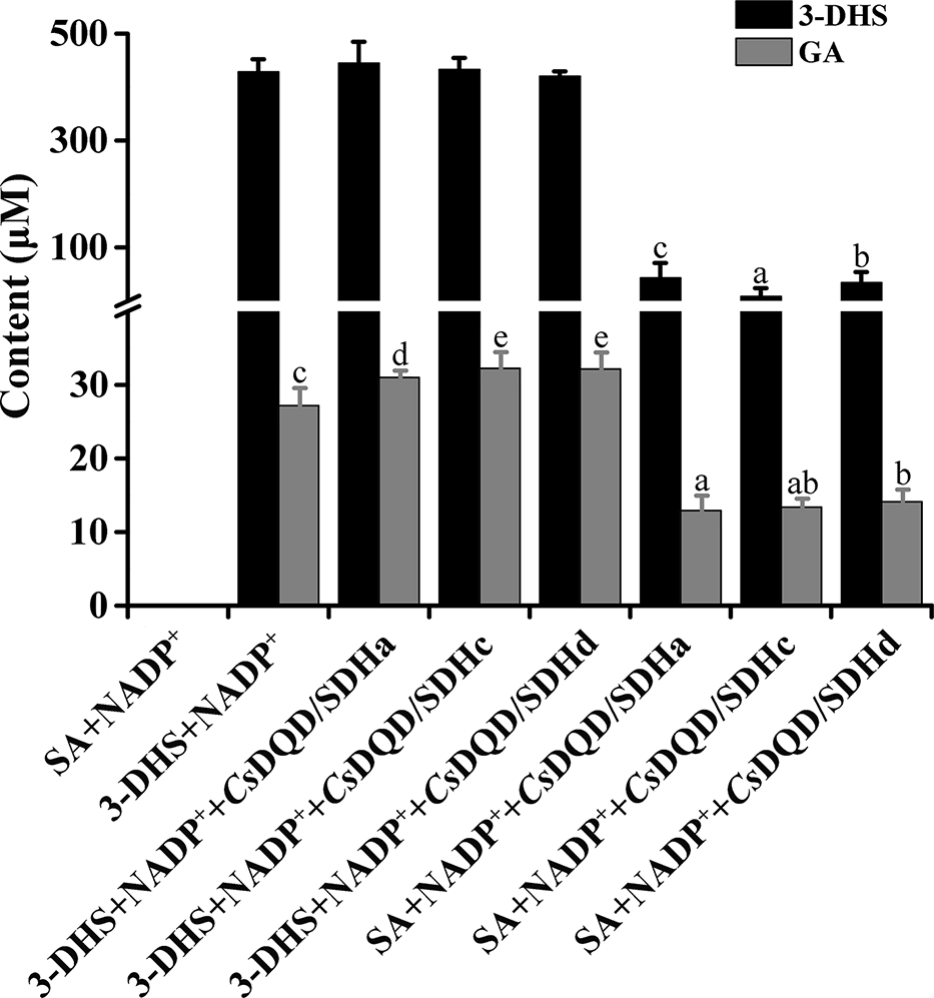
GA generation capacity from *Cs*DQD/SDHa, *Cs*DQD/SDHc, and *Cs*DQD/SDHd and without enzyme as the control. The reaction products in the enzymatic assays were quantified using HPLC (λ_3-DHS_ = 234 nm, λ_GA_ = 280 nm). Data are shown as the mean of three replicates ± SD. Different letters represent statistically differ at *P* < 0.05 according to one-way ANOVA of Duncan’s test. GA, gallic acid; HPLC, high-performance liquid chromatography; ANOVA, analysis of variance.

GA production was found in *Cs*DQD/SDHs assays. SA generation occurred immediately in the 3-DHS reduction reaction catalyzed by *Cs*DQD/SDHa and reached its maximum value after 15 min, whereas GA was detected after 20 min in time course of the reaction (**Figure 4A**). A similar pattern was observed for SA oxidation by *Cs*DQD/SDHa; the increase in GA content was consistent with the decrease in the product 3-DHS (**Figure 4B**). On the basis of these results, we speculate that GA may be directly generated from 3-DHS with NADP^+^ as the coenzyme. However, a significant increase in GA production was not detected in the time course of the *Cs*DQD/SDHa assay when using 3-DHS and the coenzyme NADP^+^ as substrates (**Figure 4C**). To further verify that GA was directly generated from 3-DHS, enzymatic or nonenzymatic *Cs*DQD/SDH assay was conducted using 3-DHS and the coenzyme NADP^+^ as substrates, and the assay revealed GA was directly generated from 3-DHS in the enzymatic SA oxidation reaction (**Figure 5**).

### Determination of Kinetic Parameters

To determine the optimum pH, assays were performed at 30°C for 30 min with citric acid buffer (100 mM, pH 4–7), BTP-HCl buffer (100 mM, pH 6–9), and sodium carbonate buffer (100 mM, pH 8–11), individually. The results showed that the 3-DHS reduction and SA oxidation activities of *Cs*DQD/SDHa, *Cs*DQD/SDHc, and *Cs*DQD/SDHd were higher in alkaline buffer than in acidic buffer (**Supplementary Figure 4**).

*In vitro*, the kinetic parameters of both substrates (3-DHS and SA) and cofactors (NADP^+^ and NADPH) were measured at 30°C for 3 min in a buffer with pH 8.5 (**Table 1**). For 3-DHS reduction, compared with *Cs*DQD/SDHc (*K*_M(3-DHS)_ = 330.933 µM) and *Cs*DQD/SDHd (*K*_M(3-DHS_ = 465.971 µM), *Cs*DQD/SDHa (*K*_M(3-DHS)_ = 286.576 µM) had the highest affinity for 3-DHS. The catalytic efficiency of *Cs*DQD/SDHa (*k*_cat_/*K*_M(3-DHS)_ = 1,412.281 S^−1^·M^−1^) was almost three times more than that of *Cs*DQD/SDHc (*k*_cat_/*K*_M(3-DHS)_ = 506.596 S^−1^·M^−1^) and 14 times more than that of *Cs*DQD/SDHd (*k*_cat_/*K*_M(3-DHS)_ = 99.546 S^−1^·M^−1^). For SA oxidation, *Cs*DQD/SDHc (*K*_M(SA)_ = 199.653 µM) showed the highest affinity for the substrate SA in comparison with *Cs*DQD/SDHa (*K*_M(SA)_ = 272.782 µM) and *Cs*DQD/SDHd (*K*_M(SA)_ = 610.643 µM). However, the catalytic efficiency of *Cs*DQD/SDHa (*k*_cat_/*K*_M(SA)_ = 562.168 S^−1^·M^−1^) and *Cs*DQD/SDHc (*k*_cat_/*K*_M(SA)_ = 535.744 S^−1^·M^−1^) were comparable and approximately four times more than that of *Cs*DQD/SDHd (*k*_cat_/*K*_M(SA)_ = 138.482 S^−1^·M^−1^). In summary, *Cs*DQD/SDHa compared with *Cs*DQD/SDHc and *Cs*DQD/SDHd had the highest catalytic efficiency for 3-DHS reduction and SA oxidation.

**Table 1:** Michaelis–Menten kinetic parameters of recombinant *Cs*DQD/SDHs for each substrate.

Enzyme	Substrate	Product	K_M_ (μM)	V_max_ (nKat·μg^-1^)	k_cat_ (S^-1^)	k_cat_/K_M_ (S^-1^·M^-1^)
***Cs*DQD/SDHa**	NADPH	SA	270.306	31.545	0.202	746.282
	3-DHS		286.576	63.291	0.405	1412.281
	NADP^+^	3-DHS	138.031	18.248	0.117	845.397
	SA		272.782	23.981	0.153	562.168
***Cs*DQD/SDHb**	NADPH	SA	nd*			
	3-DHS		nd			
	NADP^+^	3-DHS	nd			
	SA		nd			
***Cs*DQD/SDHc**	NADPH	SA	267.244	7.987	0.052	193.409
	3-DHS		330.933	25.907	0.168	506.596
	NADP^+^	3-DHS	114.553	6.579	0.043	371.655
	SA		199.653	16.529	0.107	535.744
***Cs*DQD/SDHd**	NADPH	SA	248.798	7.758	0.049	196.708
	3-DHS		465.971	7.353	0.046	99.546
	NADP^+^	3-DHS	46.342	0.937	0.006	127.616
	SA		610.643	13.405	0.085	138.482

nd, no activity detectable or too low to determine kinetic properties.

Notably, *Cs*DQD/SDHs showed considerable individual differences between the catalytic efficiency of 3-DHS reduction and SA oxidation; *Cs*DQD/SDHa had higher catalytic efficiency for 3-DHS reduction than for SA oxidation, *Cs*DQD/SDHd showed the opposite tendency, and *Cs*DQD/SDHc had almost equal catalytic efficiency for 3-DHS reduction and SA oxidation. In addition, *Cs*DQD/SDHd had higher affinity for NADP^+^. These results suggest that *Cs*DQD/SDHc and *Cs*DQD/SDHd function differently in tea plants, such as efficient GA generation.

### Truncation and Site-Directed Mutagenesis

**Figure 6:**
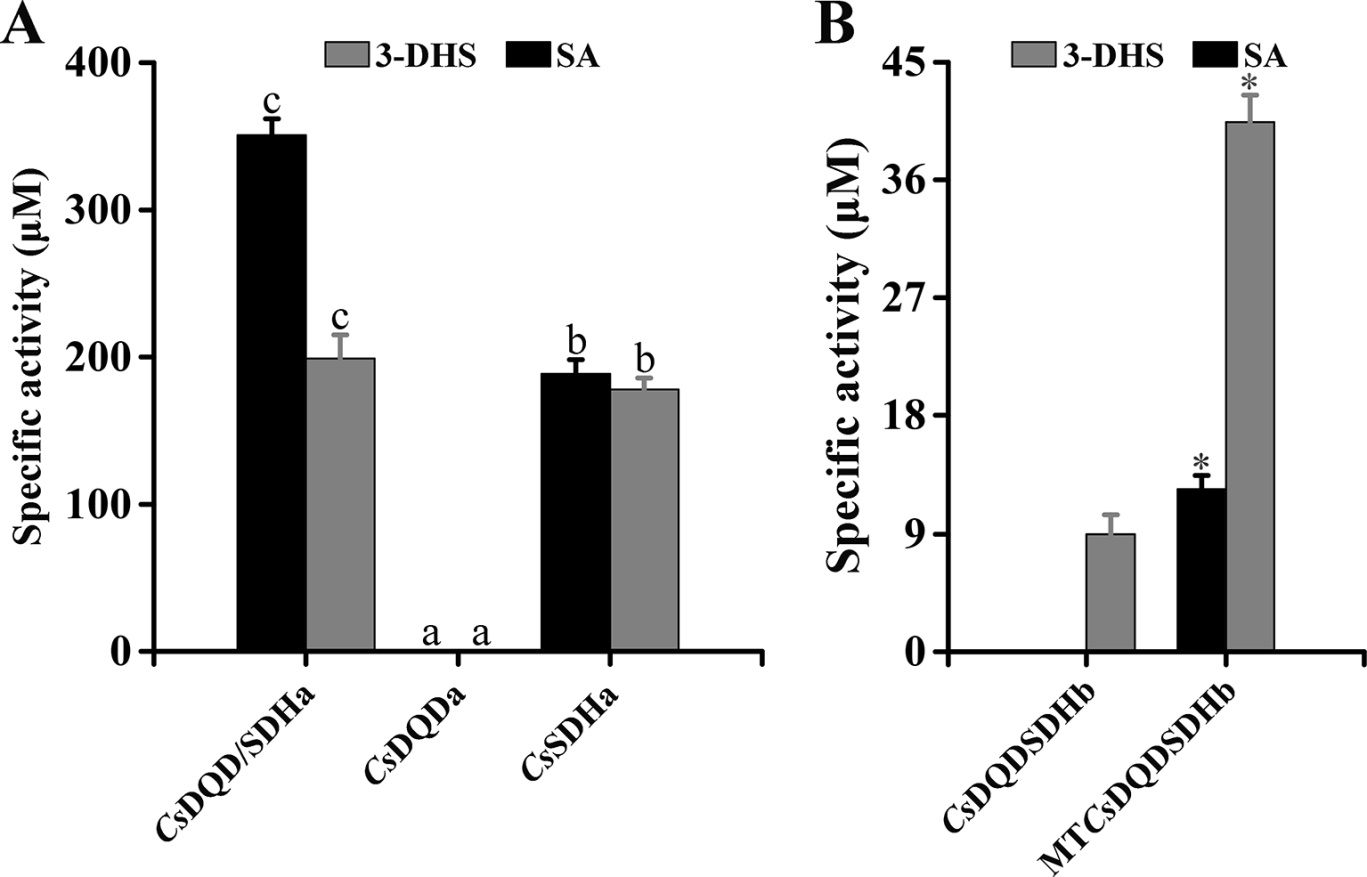
Effect of key unit and site-directed mutagenesis on the activity of *Cs*DQD/SDHa and *Cs*DQD/SDHb, respectively. **(A)** Specific activity analysis of the *Cs*DQDa and *Cs*SDHa truncated mutant proteins. **(B)** Specific activity analysis of the purified MT*Cs*DQD/SDHb mutant protein. Data are presented as the means of three independent assays. Data are shown as the mean of three replicates ± SD. Different letters in **(A)** are statistically different at *P* < 0.05 according to one-way ANOVA of Duncan’s test. Asterisks in **(B)** indicate significant difference based on Tukey’s test (*P* < 0.05). ANOVA, analysis of variance.

To determine the function of the SDH unit and the DQD unit, two units were expressed in *Escherichia coli*, respectively, and the recombinant proteins were named *Cs*DQDa and *Cs*SDHa. The results of the enzyme assay indicated that compared with the *Cs*DQD/SDHa recombinant protein, the *Cs*SDHa recombinant protein had an almost identical enzymatic activity for NADPH-dependent reduction of 3-DHS and a lower enzymatic activity for NADP^+^-dependent oxidation of SA, whereas the *Cs*DQDa recombinant protein was completely inactive (**Figure 6A**).

The sequence alignments in **Supplementary Figure 2** showed that the key residues Ser-338 and NRT in the SDH unit of *Cs*DQD/SDHb were replaced by Gly and DI/LD, respectively; this may be a reason that the enzymatic activities of DQD/SDH proteins in the second group were difficult to detect. To verify this postulation, the substrate and cofactor binding sites of *Cs*DQD/SDHb were analyzed using site-directed mutagenesis. Referring to the model of *A. thaliana* protein crystal structure, the residue sites of Gly-338, Gly-381, Asp-483, Leu-484, and Asp-485 in *Cs*DQD/SDHb were mutated to Ser-338, Thr-381, Asn-483, Arg-484, and Thr-485, respectively; the mutant protein was named MT*Cs*DQD/SDHb. The results showed that MT*Cs*DQD/SDHb had a similar reduction activity of 3-DHS and had six times higher oxidation activity of SA than *Cs*DQD/SDHb (**Figure 6B**), suggesting that the mutation of residues Ser-338 and NRT to Gly and DI/LD in the SDH unit is the reason for the low activity of *Cs*DQD/SDHb, respectively.

### Expression Pattern of CsDQD/SDHs and Accumulation of GA, βG, ECG, and EGCG in Tea Plants

**Figure 7:**
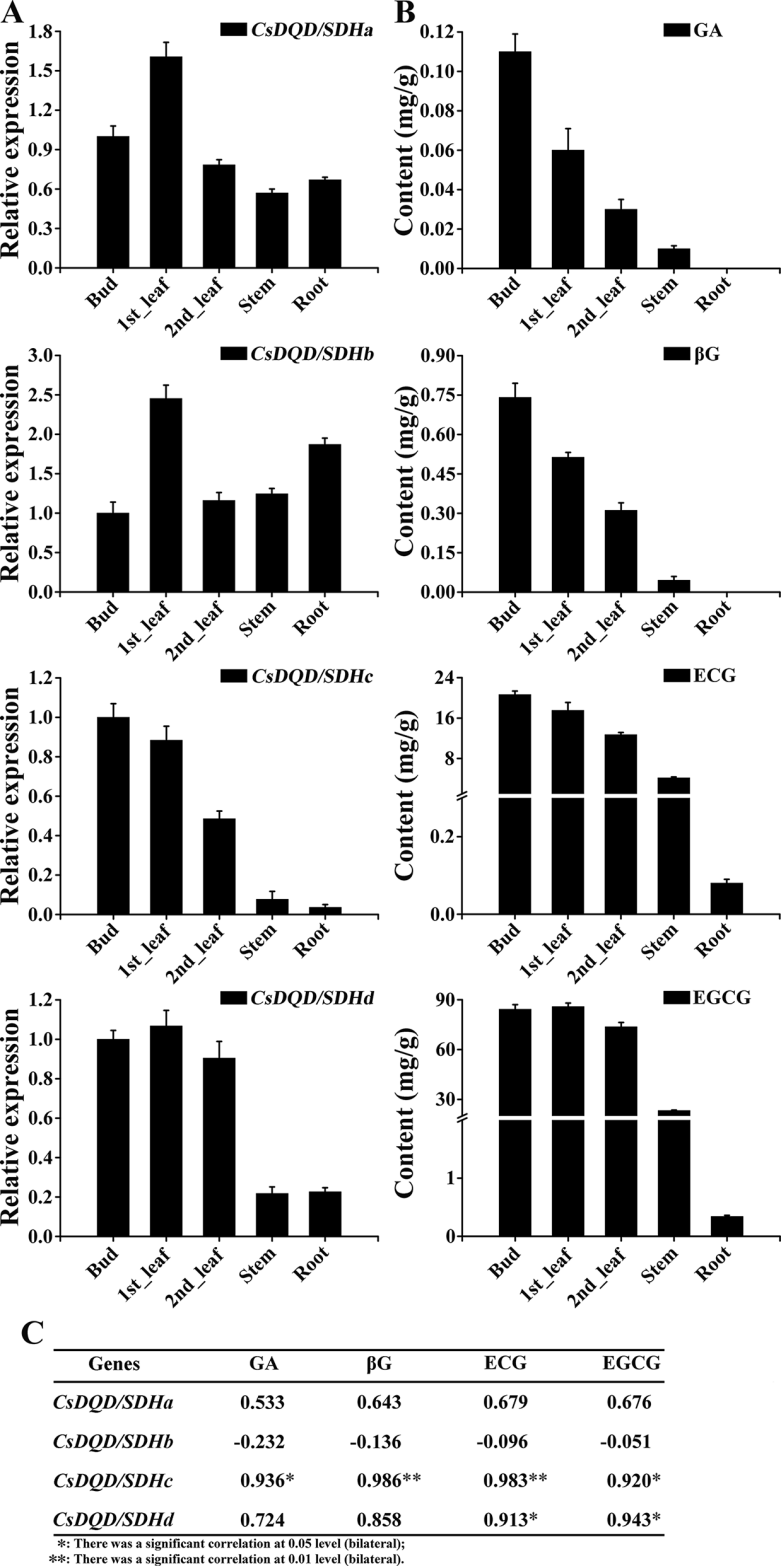
Expression patterns of four *CsDQD/SDH* genes and the accumulation profiles of GA and its derivatives in different tissues of tea plants. **(A)** Expression profiles of the *CsDQD/SDHa*, *CsDQD/SDHb*, *CsDQD/SDHc*, and *CsDQD/SDHd* genes in different organs. **(B)** Quantitative analysis of GA, βG, ECG, and EGCG contents in different tea organs. Data are presented as the means of three independent assays. **(C)** Analysis of the Pearson correlation coefficient of the expression pattern of *CsDQD/SDHs* and the content of GA, βG, ECG, and EGCG. GA, gallic acid; ECG, epicatechin gallate; EGCG, epigallocatechin gallate.

The relative expression pattern of the four *CsDQD/SDHs* in diverse plant parts (bud, first leaf, second leaf, stem, and root) was determined using qRT-PCR (**Figure 7A**). The results revealed that *CsDQD/SDHa* and *CsDQD/SDHb* showed similar expression patterns in buds, young leaves, stems, and roots and showed the highest expression in first leaves. *CsDQD/SDHc* and *CsDQD/SDHd* were highly expressed in the bud and tender leaves, and their expression decreased from the bud to the root, with the least expression in roots.

The contents of GA and its glucose ester, βG, were higher in buds and young leaves than in other tissues. A positive correlation was observed between the expression pattern of *CsDQD/SDHc* and *CsDQD/SDHd* and the accumulation of GA, βG, ECG, and EGCG (**Figure 7B**). The Pearson correlation coefficient (**Figure 7C**) indicating that the expression pattern of *CsDQD/SDHc* was more positively related to the synthesis of GA, βG, and galloylated catechins.

## Discussion

Early studies have also uncovered the importance of the SA pathway in plant secondary compound synthesis, including lignin and pigments such as anthocyanins. Research related to the key gene involved in GA biosynthesis in tea plants is scant but indicates that *DQD/SDH*s are the candidate target genes. GA serves as a precursor for the biosynthesis of galloyl-type HTs and galloylated PAs (Haslam and Cai, 1994). HTs play crucial roles in tea plant herbivore deterrence and influence tea’s bitterness and astringency. A critical step in HT production is βG generation. For βG formation, *Cs*UGT84A22 requires GA as a substrate (Cui et al., 2016), which is produced from 3-DHS of the SA pathway. In addition, UGT84A23, UGT84A24, and DQD/SDH are localized in the cytoplasm in *Punica granatum* (Ono, 2016); therefore, it is conceivable that GA production in cytosolic from *Cs*DQD/SDHs may be supplied to *Cs*UGT84A22-catalyzed reactions. PAs, also known as condensed tannins, are polymers of flavan-3-ol units such as catechins and galloylated catechins (Winkel-Shirley, 2001). The galloylated catechins EGCG and PA polymers are the dominant flavonoids in tea leaves and roots, respectively (Jiang et al., 2013; Jiang et al., 2015). Previous studies have shown that a UDP-glycosyltransferases and a serine carboxypeptidase-like (SCPL) acyltransferase participate in the biosynthesis of galloylated flavanol-3-ol and galloyl-type HTs (Gross, 2008).

In plants, DQD/SDH is an essential bifunctional enzyme involved in the SA pathway producing chorismic acid, which is converted into several secondary metabolites (Herrmann, 1995; Maeda and Dudareva, 2012). As a bi-functional or tri-functional enzyme, the DQD/SDH protein plays vital roles in controlling 3-DHS reduction, SA oxidation, and GA synthesis. Plant *DQD/SDH* belongs to the multigene family and may play different roles in 3-DHS reduction, SA oxidation, and GA synthesis (Kasama et al., 2007). Enzyme–substrate kinetic analysis indicated that *Cs*DQD/SDHa had higher catalytic efficiency for 3-DHS reduction than for SA oxidation; therefore, we can assume that *Cs*DQD/SDHa directs carbon flux towards aromatic acids and biosynthesis in the main trunk of the SA pathway, effectively converting 3-DHS into SA. Ding et al. revealed that RNAi-mediated reduction of NtDHD/SHD-1 (in the first group with *Cs*DQD/SDHa) expression resulted in severe metabolic and phenotypic alterations in tobacco plants (Kasama et al., 2007), highlighting the essentiality of DQD/SDHs (from the first group) for plant growth and development. *Cs*DQD/SDHd showed the opposite tendency compared with *Cs*DQD/SDHa, whereas *Cs*DQD/SDHc had almost equal catalytic efficiency for 3-DHS reduction and SA oxidation. All these findings imply that *Cs*DQD/SDHc and *Cs*DQD/SDHd have different functions compared with *Cs*DQD/SDHa *in vivo*; for instance, they might be more suitable for SA oxidation and result in continuous GA generation. In grape berry, the highest expression of *Vv*SDH3 and *Vv*SDH4 occurred in parallel with galloylated PA accumulation (Bogs et al., 2005; Kennedy et al., 2010). *Cs*DQD/SDHc and *Cs*DQD/SDHd generate high amounts of galloylated flavan-3-ols in tea plants (Jiang et al., 2013). Moreover, the phenomenon of the consistency of the expression pattern of *CsDQD/SDHc* and *CsDQD/SDHd* with the accumulation pattern of GA, *βG*, gallocatechin gallate (GCG), and EGCG in tea plants showed that the *CsDQD/SDHc* and *CsDQD/SDHd* genes are involved in GA synthesis. In addition, *Dk*SDH, which clustered with *Cs*DQD/SDHd into the fourth group, was downregulated in nonastringent persimmon fruits (with low galloylated PA levels) compared with astringent persimmon fruits (Ikegami et al., 2005).

The function of *Cs*DQD/SDH in GA synthesis remains unclear. GA could be spontaneously generated from 3-DHS in the enzymatic or nonenzymatic *Cs*DQD/SDHs assay when using 3-DHS and the coenzyme NADP^+^ as substrates.

Plant DQD/SDH belongs to the second group, which is unique. In tea plants, among the four proteins, the *Cs*DQD/SDHa, *Cs*DQD/SDHc, and *Cs*DQD/SDHd proteins exhibited a mainstream activity for the substrates 3-DHS and SA with the cofactors of NADPH and NADP^+^, respectively, whereas *Cs*DQD/SDHb exhibited a very low mainstream activity. Site-directed mutagenesis suggested that mutation of residues Ser-338 and NRT to Gly and DI/LD in the SDH unit, respectively, is the reason for the low activity of *Cs*DQD/SDHb for 3-DHS reduction and SA oxidation. Notably, the Poptr2 and Poptr3 proteins from *P. trichocarpa* belonging to the second group quinate dehydrogenase (QDH) activity; these proteins are involved in the synthesis of quinate and its derivatives (Guo et al., 2014). GQA, a conjugate of quinic acid (QA) and GA, highly accumulates in tea plants (Jiang et al., 2013). Chlorogenic acid, a QA derivative, is involved in antimicrobial and antiherbivore activities, and it mainly accumulates in the roots of carrot (Cole, 2010b), sweet potato (Mcclure, 1960), and lettuce plants (Cole, 2010a); it is induced by microbe and herbivore infestation. Because of its high sequence identity with Poptr2 and Poptr3, *Cs*DQD/SDHb must be a favorable candidate enzyme for the biosynthesis of QA and its derivatives, and a study examining this postulation is ongoing.

In conclusion, experimental evidence from enzyme assays and kinetic analysis indicates that *CsDQD/SDHc* and *CsDQD/SDHd* are favorable candidate genes for GA biosynthesis in tea plants. This study advances our understanding of GA, and its metabolism is tightly connected to the SA pathway.

## Data Availability Statement

All datasets for this study are included in the manuscript/**Supplementary Files**.

## Author Contributions

KH, ML, LG, and TX conceived and designed research. KH, ML, YL, and YZ performed the real-time PCR experiments. KH, MZ and GZ analyzed the data. YW, LZ, and XD revised the English writing of the manuscript. YW, LZ, and LG revised the English writing of the manuscript. All authors read and approved the manuscript.

## Funding

This work was supported by the National Natural Science Foundation of China (31870676, 31570694, 31470689), National Key Research and Development Program of China (2018YFD1000601), and the Natural Science Foundation of Anhui Province, China (1908085MC100).

## Conflict of Interest

The authors declare that the research was conducted in the absence of any commercial or financial relationships that could be construed as a potential conflict of interest.

## References

[B1] Akagi T. Ikegami A. Suzuki Y. Yoshida J. Yamada M. Sato A. (2009). Expression balances of structural genes in shikimate and flavonoid biosynthesis cause a difference in proanthocyanidin accumulation in persimmon (*Diospyros kaki Thunb.*) fruit. Planta 230 (5), 899–915. 10.1007/s00425-009-0991-6 19669159

[B2] Bentley R. Haslam E. (2008). The shipmate pathway—a metabolic tree with many branches. Ccr Crit. Rev. Biochem. 25, 307–384. 10.3109/10409239009090615 2279393

[B3] Bogs J. Downey M. O. Harvey J. S. Ashton A. R. Tanner G. J. Robinson S. P. (2005). Proanthocyanidin synthesis and expression of genes encoding leucoanthocyanidin reductase and anthocyanidin reductase in developing grape berries and grapevine leaves. Plant Physiol. 139 (2), 652–663. 10.1104/pp.105.064238 16169968 PMC1255985

[B4] Bontpart T. Marlin T. Vialet S. Guiraud J. L. Pinasseau L. Meudec E.(2016). Two shikimate dehydrogenases, VvSDH3 and VvSDH4, are involved in gallic acid biosynthesis in grapevine. J. Exp. Bot. 67 (11), 3537–3550. 10.1093/jxb/erw184 27241494 PMC4892741

[B5] Cole R. A. (2010a). Phenolic acids associated with the resistance of lettuce cultivars to the lettuce root aphid. Ann. Appl. Biol. 105 (1), 129–145. 10.1111/j.1744-7348.1984.tb02809.x

[B6] Cole R. A. (2010b). Relationship between the concentration of chlorogenic acid in carrot roots and the incidence of carrot fly larval damage. Ann. Appl. Biol. 106 (2), 211–217. 10.1111/j.1744-7348.1985.tb03110.x

[B7] Cui L. Yao S. Dai X. Yin Q. Liu Y. Jiang X. (2016). Identification of UDP-glycosyltransferases involved in the biosynthesis of astringent taste compounds in tea (*Camellia sinensis*). J. Exp. Bot. 67 (8), 2285–2297. 10.1093/jxb/erw053 26941235 PMC4809296

[B8] Dewick P. M. Haslam E. (1969). Phenol biosynthesis in higher plants. Gallic acid. Biochem. J. 113 (3), 537. 10.1042/bj1130537 5807212 PMC1184696

[B9] Dyer W. E. Henstrand J. M. Handa A. K. Herrmann K. M. (1989). Wounding induces the first enzyme of the shikimate pathway in Solanaceae. Proc. Nat. Acad. Sci. U.S.A. 86 (19), 7370–7373. 10.1073/pnas.86.19.7370 PMC298063 16594071

[B10] Fiedler E. Schultz G. (1985). Localization, purification, and characterization of shikimate oxidoreductase-dehydroquinate hydrolyase from stroma of spinach chloroplasts. Plant Physiol. 79 (1), 212–218. 10.1104/pp.79.1.212 16664373 PMC1074854

[B11] Gross G. G. (2008). From lignins to tannins: Forty years of enzyme studies on the biosynthesis of phenolic compounds. Phytochemistry 69 (18), 3018–3031. 10.1016/j.phytochem.2007.04.031 17559893

[B12] Guo J. Carrington Y. Alber A. Ehlting J. (2014). Molecular characterization of quinate and shikimate metabolism in *Populus trichocarpa*. J. Biol. Chem. 289 (34), 23846–23858. 10.1074/jbc.M114.558536 24942735 PMC4156088

[B13] Haslam E. Cai Y. (1994). Plant polyphenols (*vegetable tannins*): gallic acid metabolism. Nat. Prod. Rep. 11 (1), 41–66. 10.1039/np9941100041 15206456

[B14] Herrmann K. M. (1995). The shikimate pathway: early steps in the biosynthesis of aromatic compounds. Plant Cell 7 (7), 907–919. 10.2307/3870046 12242393 PMC160886

[B15] Ikegami A. Yonemori K. Kitajima A. Sato A. Yamada M. (2005). Expression of genes involved in proanthocyanidin biosynthesis during fruit development in a Chinese pollination-constant, nonastringent (PCNA) persimmon, ‘Luo Tian Tian Shi’. J. Am. Soc. Hortic. Sci. Am. Soc. Hortic. Sci. 41 (130), 830. 10.21273/JASHS.130.6.830

[B16] Ishikura N. Hayashida S. Tazaki K. (1984). Biosynthesis of gallic and ellagic acids with 14C-labeled compounds in *Acer* and *Rhus* leaves. Bot. Mag. = Shokubutsu-gaku-zasshi 97 (3), 355–367. 10.1007/BF02488668

[B17] Jiang X. Liu Y. Li W. Zhao L. Meng F. Wang Y. (2013). Tissue-specific, development-dependent phenolic compounds accumulation profile and gene expression pattern in tea plant [*Camellia sinensis*]. Plos One 8 (4), e62315. 10.1371/journal.pone.0062315 23646127 PMC3639974

[B18] Jiang X. Liu Y. Wu Y. Tan H. Meng F. Wang Y. S. (2015). Analysis of accumulation patterns and preliminary study on the condensation mechanism of proanthocyanidins in the tea plant [*Camellia sinensis*]. Sci. Rep. 5, 8742. 10.1038/srep08742 25735226 PMC4348662

[B19] Kasama J. S. T. Ohishi M. Ding L. Hofus D. Hajirezaei M-R. Ferni A. R. (2007). Functional analysis of the essential bifunctional tobacco enzyme 3-dehydroquinate dehydratase/shikimate dehydrogenase in transgenic tobacco plants. J. Exp. Bot. 58, 2053–2067. 10.1093/jxb/erm059 17463052

[B20] Kennedy J. A. Troup G. J. Pilbrow J. R. Hutton D. R. Hewitt D. Hunter C. R. (2010). Development of seed polyphenols in berries from *Vitis vinifera L. cv. Shiraz*. Aust. J. Grape Wine Res. 6 (3), 244–254. 10.1111/j.1755-0238.2000.tb00185.x

[B21] Liu Y. Gao L. Liu L. Yang Q. Lu Z. Nie Z. (2012). Purification and characterization of a novel galloyltransferase involved in catechin galloylation in the tea plant. J. Biol. Chem. 287. 10.1074/jbc.M112.403071 PMC3531754 23132863

[B22] Maeda H. Dudareva N. (2012). The shikimate pathway and aromatic amino acid biosynthesis in plants. Annu. Rev. Plant Biol. 63, 73–105. 10.1146/annurev-arplant-042811-105439 22554242

[B23] Mcclure T. T. (1960). Chlorogenic acid accumulation and wound healing in sweet potato roots. Am. J. Bot. 47 (4), 277–280. 10.2307/2439607

[B24] Michel G. Roszak A. W. Sauvé V. Maclean J. Matte A. Coggins J. R. (2003). Structures of shikimate dehydrogenase AroE and its paralog YdiB. A common structural framework for different activities. J. Biol. Chem. 278 (21), 19463. 10.1074/jbc.M300794200 12637497

[B25] Muir R. M. Ibáñez A. M. Uratsu S. L. Ingham E. S. Leslie C. A. Mcgranahan G. H. (2011). Mechanism of gallic acid biosynthesis in bacteria (*Escherichia coli*) and walnut (*Juglans regia*). Plant Mol. Biol. 75 (6), 555–565. 10.1007/s11103-011-9739-3 21279669 PMC3057006

[B26] Nagpala E. G. Guidarelli M. Gasperotti M. Masuero D. Bertolini P. Vrhovsek U. (2016). Polyphenols variation in fruits of the susceptible strawberry cultivar Alba during ripening and upon fungal pathogen interaction and possible involvement in unripe fruit tolerance. J. Agric. Food Chem. 64, 1869–1878. 10.1021/acs.jafc.5b06005 26895094

[B27] Niemetz R. Gross G. G. (2005). Enzymology of gallotannin and ellagitannin biosynthesis. Phytochemistry 66 (17), 2001–2011. 10.1016/j.phytochem.2005.01.009 16153405

[B28] Ono N. N. Xiaoqiong Q. Wilson A. E. Gang L. Li T. (2016). Two UGT84 family glycosyltransferases catalyze a critical reaction of hydrolyzable tannin biosynthesis in pomegranate (Punica granatum). PLoS One 1–25. 10.1371/journal.pone.0156319 PMC4882073 27227328

[B29] Ossipov V. Salminen J. P. Ossipova S. E. Pihlaja K. (2003). Gallic acid and hydrolysable tannins are formed in birch leaves from an intermediate compound of the shikimate pathway. Biochem. Syst. Ecol. 31 (1), 3–16. 10.1016/S0305-1978(02)00081-9

[B30] Padyana A. K. Burley S. K. (2003). Crystal structure of shikimate 5-dehydrogenase (SDH) bound to NADP: insights into function and evolution. Structure 11 (8), 1005–1013. 10.1016/S0969-2126(03)00159-X 12906831

[B31] Singh C. A. (2006). Structure of *Arabidopsis* dehydroquinate dehydratase-shikimate dehydrogenase and implications for metabolic channeling in the shikimate pathway†,‡. Biochemistry 45 (25), 7787. 10.1021/bi060366 16784230

[B32] Sprenger G. A. (2006). Aromatic amino acids. (Microbiol Monogr) 93–127 10.1007/7171_2006_067

[B33] Steinmann J. Buer J. Pietschmann T. Steinmann E. (2013). Anti-infective properties of epigallocatechin-3-gallate (EGCG), a component of green tea. Br. J. Pharmacol. 168 (5), 1059–1073. 10.1111/bph.12009 23072320 PMC3594666

[B34] Tamura K. Peterson D. Peterson N. Stecher G. Nei M. Kumar S. (2011). MEGA5: molecular evolutionary genetics analysis using maximum likelihood, evolutionary distance, and maximum parsimony methods. Mol. Biol. Evol. 28 (10), 2731. 10.1007/0-306-48380-7_2546 21546353 PMC3203626

[B35] Vit K. Katerina K. Zuzana R. Kamil K. Daniel J. Ludek J. (2008). Condensed and hydrolysable tannins as antioxidants influencing the health. Mini Rev. Med. Chem. 8 (5), 436–447. 10.2174/138955708784223486 18473933

[B36] Vogan E. (2003). Shikimate dehydrogenase structure reveals novel fold. Structure 11 (8), 902–903. 10.1016/S0969-2126(03)00165-5 12906820

[B37] Wang P. Zhang L. Jiang X. Dai X. Xu L. Li T. (2018). Evolutionary and functional characterization of leucoanthocyanidin reductases from *Camellia sinensis* . Planta 247 (1), 139–154. 10.1007/s00425-017-2771-z 28887677 PMC5756577

[B38] Winkel-Shirley B. (2001). Flavonoid biosynthesis. A colorful model for genetics, biochemistry, cell biology, and biotechnology. Plant Physiol. 126 (2), 485–493. 10.1104/pp.126.2.485 11402179 PMC1540115

[B39] Xu F. Cao S. F. Shi L. Y. Chen W. Su X. G. Yang Z. F. (2014). Blue light irradiation affects anthocyanin content and enzyme activities involved in postharvest strawberry fruit. J. Agric. Food Chem. 62, 4778–4783. 10.1021/jf501120u 24783962

[B40] Ye S. Von D. F. Brooun A. Knuth M. W. Swanson R. V. Mcree D. E. (2003). The crystal structure of shikimate dehydrogenase (AroE) reveals a unique NADPH binding mode. J. Bacteriol. 185 (14), 4144. 10.1128/JB.185.14.4144-4151.2003 12837789 PMC164887

[B41] Zhao X. Wang P. Li M. Wang Y. Jiang X. Cui L. (2017). Functional characterization of a new tea (*Camellia sinensis*) flavonoid glycosyltransferase. J. Agric. Food Chem. 65 (10), 2074. 10.1021/acs.jafc.6b05619 28220704

