# Supplementary figures and images for: Functional Analysis of 3-Dehydroquinate Dehydratase/Shikimate Dehydrogenases Involved in Shikimate Pathway in Camellia sinensis
Source: Front Plant Sci. 2019 Oct 11;10:1268. doi: 10.3389/fpls.2019.01268 (PMC6797610; doi:10.3389/fpls.2019.01268)

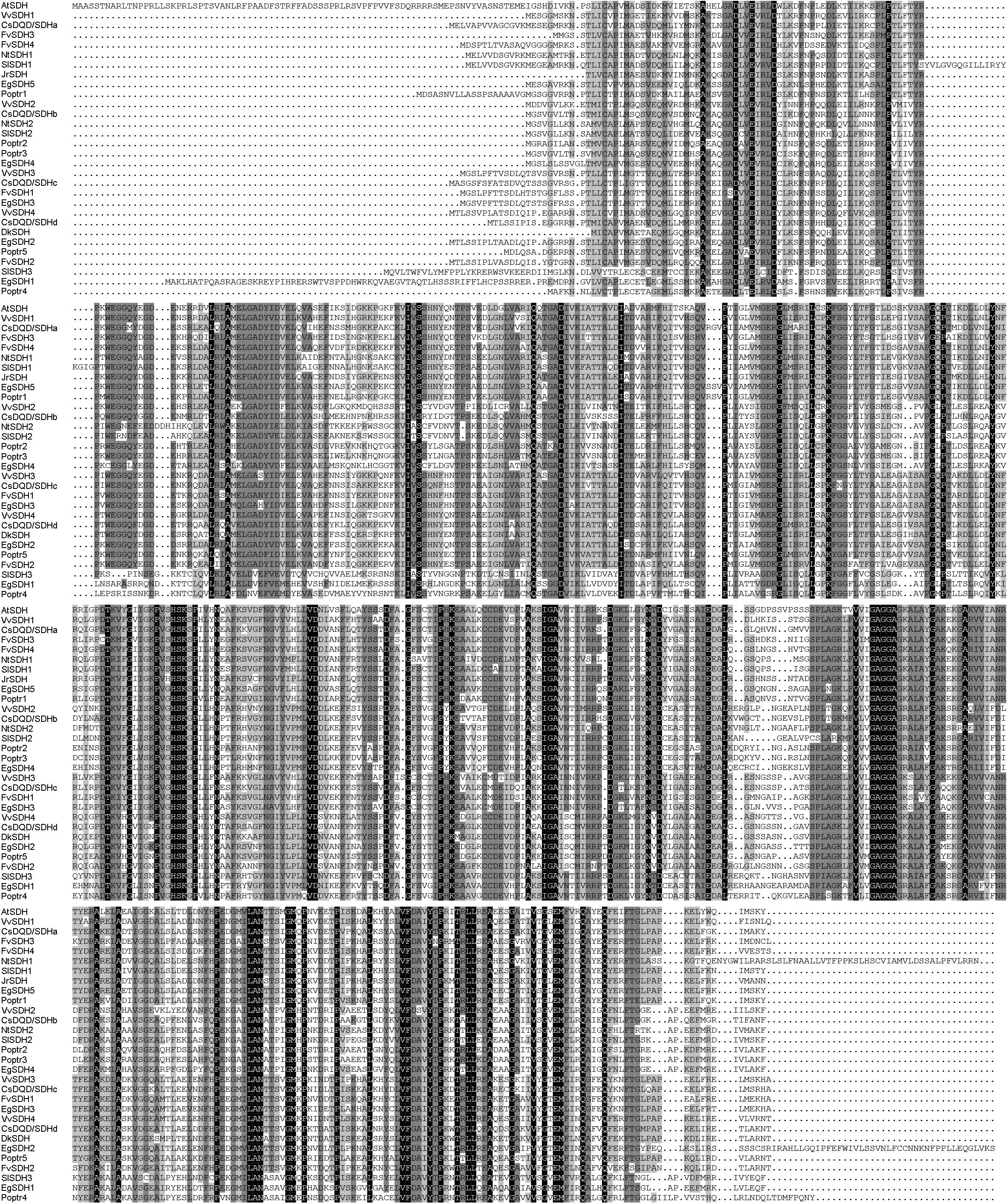

Supplement: Supplementary file 1 [file Image_1.tif]

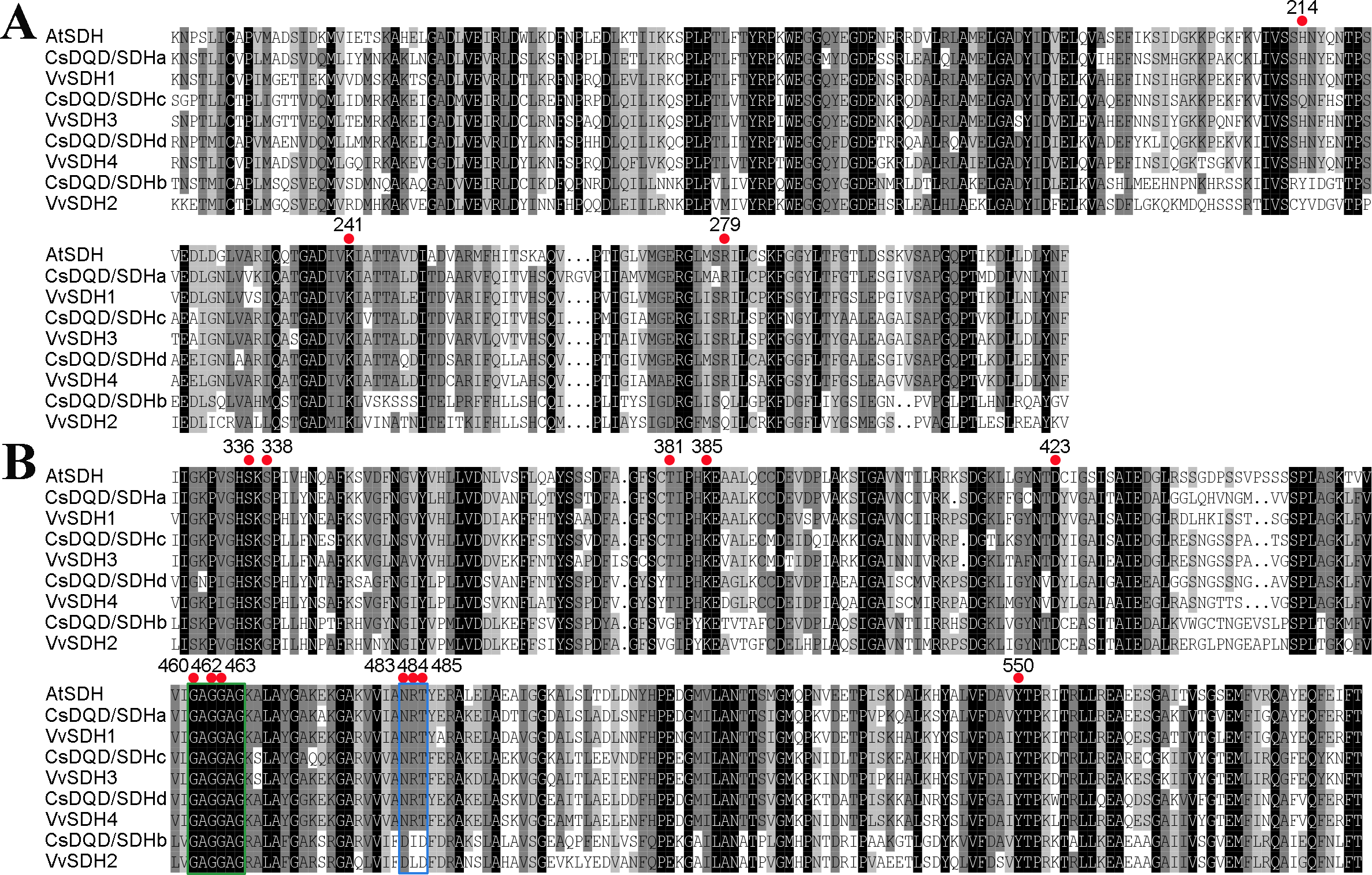

Supplement: Supplementary file 2 [file Image_2.tif]

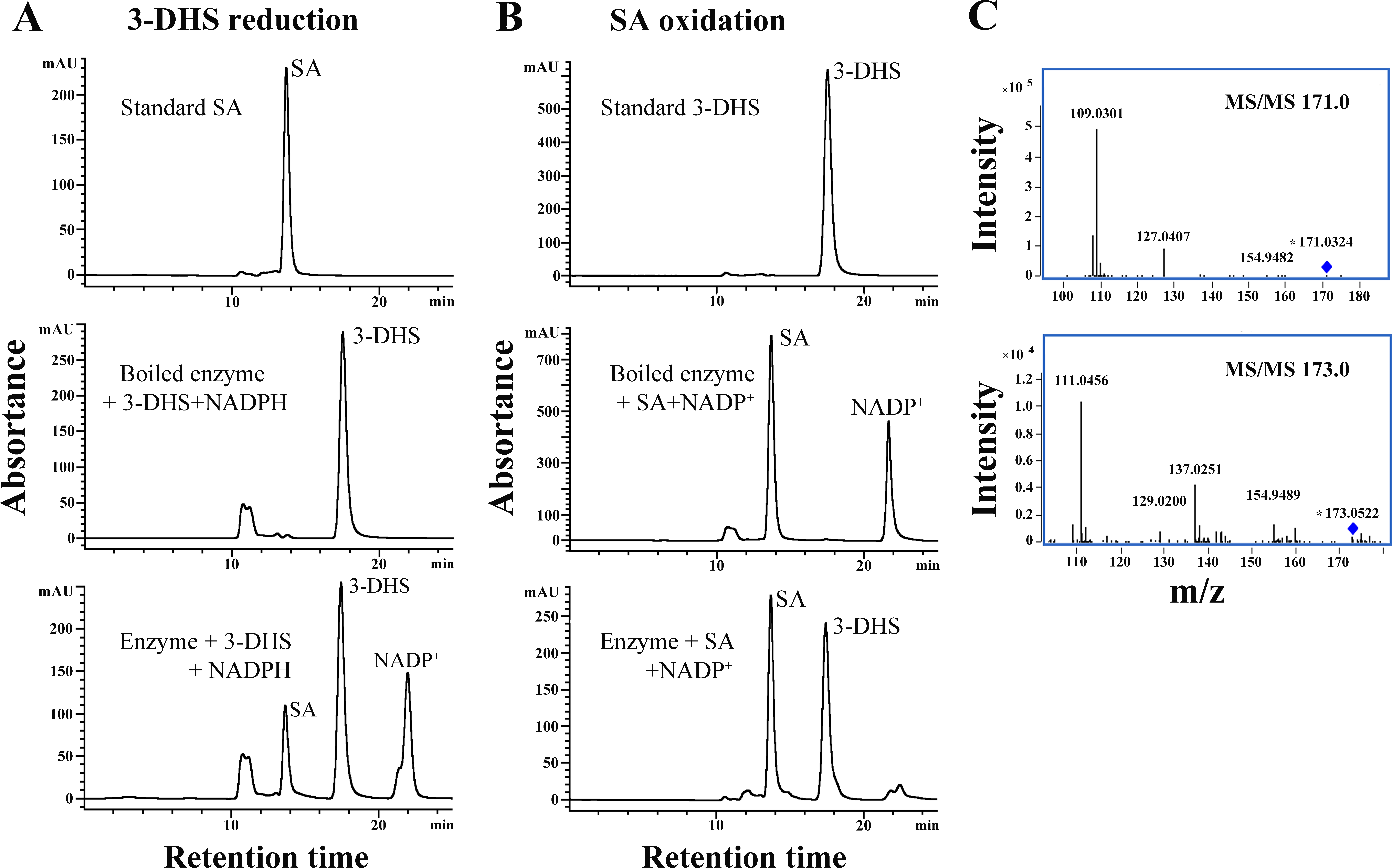

Supplement: Supplementary file 3 [file Image_3.jpeg]

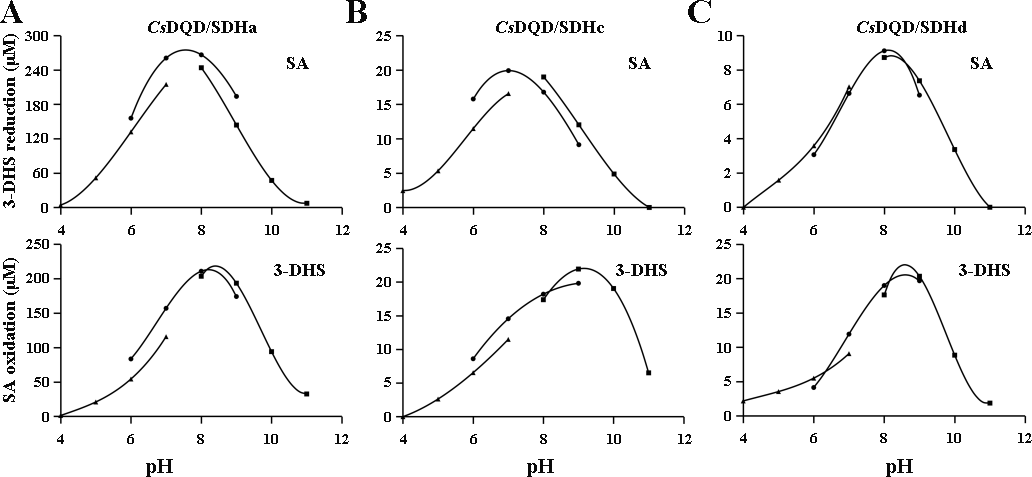

Supplement: Supplementary file 4 [file Image_4.tif]
