# Supplementary material for: Functional Analysis of 3-Dehydroquinate Dehydratase/Shikimate Dehydrogenases Involved in Shikimate Pathway in Camellia sinensis
Source: Front Plant Sci. 2019 Oct 11;10:1268. doi: 10.3389/fpls.2019.01268 (PMC6797610; doi:10.3389/fpls.2019.01268)
Supplement: Supplementary file 5 [file Table_1.doc]

**Supplementary Table S1** The primer sequences used in this study.

| Purpose | Name | 5’-3’ sequence | Restriction site |
| --- | --- | --- | --- |
| Protein  expression | *Cs*DQD/SDHa-pMAL-F | GGATCCATGGAGTTGGTTGCACCT | BamHI |
|  | *Cs*DQD/SDHa-pMAL-R | CTGCAGTCAATATTTTGCCATGAT | PstI |
|  | *Cs*DQD/SDHb-pMAL-F | GGATCCATGGGTAGTGTTGGGGTG | BamHI |
|  | *Cs*DQD/SDHb-pMAL-R | CTGCAGTCAGAAGTTTGCGAAAAT | PstI |
|  | *Cs*DQD/SDHc-pMAL-F | GGATCCATGGCCTCTGGAAGCTTTTCG | BamHI |
|  | *Cs*DQD/SDHc-pMAL-R | CTGCAGCTATGCGTGTCTTGACATG | PstI |
|  | *Cs*DQD/SDHd-pMAL-F | GGATCCATGACTCTCAGCAGCAT | BamHI |
|  | *Cs*DQD/SDHd-pMAL-R | CTGCAGTTATGTGTTCTTTGCTA | PstI |
| qPCR | *Cs*DQD/SDHa-F | ACAACCGCCTTGGACATCAC |  |
|  | *Cs*DQD/SDHa-R | CCATCAAACCCCTTTCACCC |  |
|  | *Cs*DQD/SDHb-F | ATCACTCCCATCTCCGCTCAC |  |
|  | *Cs*DQD/SDHb-R | GACAACCCTCGCTCCTCTG |  |
|  | *Cs*DQD/SDHc-F | GTAGTAGCGGAGTTAGGAGCG |  |
|  | *Cs*DQD/SDHc-R | GAAGATCAGGGCGTGGATTG |  |
|  | *Cs*DQD/SDHd-F | GACAGGCTGCATTGCGTCAG |  |
|  | *Cs*DQD/SDHd-R | GGCCCCTTTCACCCATAACA |  |
|  | GAPDH-F | TTGGCATCGTTGAGGGTCT |  |
|  | GAPDH-R | CAGTGGGAACACGGAAAGC |  |
| MT*Cs*DQD/SDHb | *Cs*DQD/SDHb-338G→S-F | GTGGGCCACAGCAAAAGTCCCCTTCTCCATAAT |  |
|  | *Cs*DQD/SDHb-338G→S-R | ATTATGGAGAAGGGGACTTTTGCTGTGGCCCAC |  |
|  | *Cs*DQD/SDHb-381G→T-F | GCAGGTTTCAGTGTAACATTTCCATACAAGGAA |  |
|  | *Cs*DQD/SDHb-381G→T-R | TTCCTTGTATGGAAATGTTACACTGAAACCTGC |  |
|  | *Cs*DQD/SDHb-483D→N-F | AGGGTTGTCATTTTTAACATTGATTTTGACAGA |  |
|  | *Cs*DQD/SDHb-483D→N-R | TCTGTCAAAATCAATGTTAAAAATGACAACCCT |  |
|  | *Cs*DQD/SDHb-484I→R-F | GTTGTCATTTTTGACCGTGATTTTGACAGAGCA |  |
|  | *Cs*DQD/SDHb-484I→R-R | TGCTCTGTCAAAATCACGGTCAAAAATGACAAC |  |
|  | *Cs*DQD/SDHb-485D→T-F | GTCATTTTTGACATTACTTTTGACAGAGCAAAG |  |
|  | *Cs*DQD/SDHb-485D→T-R | CTTTGCTCTGTCAAAAGTAATGTCAAAAATGAC |  |
| *Cs*DQDa | *Cs*DQDa-F | GGATCCAAGAACTCAACCCTAATTTGT |  |
|  | *Cs*DQDa-R | CTGCAGGATATTGTACAAGTTCACCAA |  |
| *Cs*SDHa | *Cs*SDHa-F | GGATCCATTATCGGGAAGCCTGTTGGCC |  |
|  | *Cs*SDHa-R | CTGCAGTCCAGTGAACCTCTCGAATTG |  |
