# Supplementary material for: Functional Analysis of 3-Dehydroquinate Dehydratase/Shikimate Dehydrogenases Involved in Shikimate Pathway in Camellia sinensis
Source: Front Plant Sci. 2019 Oct 11;10:1268. doi: 10.3389/fpls.2019.01268 (PMC6797610; doi:10.3389/fpls.2019.01268)
Supplement: Supplementary file 6 [file DataSheet_1.pdf]

Supplementary Data

Supplementary Figure 1 Sequence consistency analysis of 30 DQD/SDH proteins from plants.

Supplementary Figure 2 Comparison of the partial DQD/SDH sequences from the tea plant and other species (*Arabidopsis* and *Vitis vinifera*) using DNAMAN 6.0. A. DQD unit; B. SDH unit. Amino acid numeration is based on the protein sequence of *A. thaliana*. The key amino acids are marked with red dots; the G-motif with a green box; and the NRT motif with a blue box. Conserved amino acids are marked with black (similarity = 100%), dark gray (similarity ≥ 75%), and light gray (similarity ≥ 50%) colors.

Supplementary Figure 3 HPLC analysis of recombinant *Cs*DQD/SDHa enzyme reaction products. (A) HPLC chromatograms from top to bottom are for the standard of SA (top), boiled enzyme reaction with 3-DHS and NADPH (middle), and enzyme production of SA (bottom); (B) HPLC chromatograms from top to bottom are for the standard of 3-DHS (top), boiled enzyme reaction with SA and $NADP^+$ (middle), and enzyme production of 3-DHS (bottom); (C) MS/MS molecular ion characteristics of the enzyme products of 3-DHS (171.0) and SA (173.0).

Supplementary Figure 4 Selection of the optimum pH value for the *Cs*DQD/SDHs reaction. A, B, and C showed the production accumulation profile at various pH values from *Cs*DQD/SDHa, *Cs*DQD/SDHc, and *Cs*DQD/SDHd, respectively. The top line shows the 3-DHS reduction reaction, the bottom line shows the SA oxidation reaction. ▲: represents the citric acid buffer; ●: represents the BTP-HCl buffer; █: represents the sodium carbonate buffer.

Supplementary Table S1 The primer sequences used in this study.

Supplementary Table S2 ID of multiple plant DQD/SDH proteins used in this study.

Purpose	Name	5'-3' sequence	Restriction site
Protein expression	*Cs*DQD/SDHa-pMAL-F	GGATCCATGGAGTTGGTTGCACCT	BamHI
	*Cs*DQD/SDHa-pMAL-R	CTGCAGTCAATATTTTGCCATGAT	PstI
	*Cs*DQD/SDHb-pMAL-F	GGATCCATGGGTAGTGTTGGGGTG	BamHI
	*Cs*DQD/SDHb-pMAL-R	CTGCAGTCAGAAGTTTGCGAAAAT	PstI
	*Cs*DQD/SDHc-pMAL-F	GGATCCATGGCCTCTGGAAGCTTTTCG	BamHI
	*Cs*DQD/SDHc-pMAL-R	CTGCAGCTATGCGTGTCTTGACATG	PstI

	*Cs*DQD/SDHd-pMAL-F	GGATCCATGACTCTCAGCAGCAT	BamHI
	*Cs*DQD/SDHd-pMAL-R	CTGCAGTTATGTGTTCTTTGCTA	PstI
qPCR	*Cs*DQD/SDHa-F	ACAACCGCCTTGGACATCAC	
	*Cs*DQD/SDHa-R	CCATCAAACCCCTTTCACCC	
	*Cs*DQD/SDHb-F	ATCACTCCCATCTCCGCTCAC	
	*Cs*DQD/SDHb-R	GACAACCCTCGCTCCTCTG	
	*Cs*DQD/SDHc-F	GTAGTAGCGGAGTTAGGAGCG	
	*Cs*DQD/SDHc-R	GAAGATCAGGGCGTGGATTG	
	*Cs*DQD/SDHd-F	GACAGGCTGCATTGCGTCAG	
	*Cs*DQD/SDHd-R	GGCCCCTTTCACCCATAACA	
	GAPDH-F	TTGGCATCGTTGAGGGTCT	
	GAPDH-R	CAGTGGGAACACGGAAAGC	
MT*Cs*DQD/SDHb	*Cs*DQD/SDHb-338G→S-F	GTGGGCCACAGCAAAAGTCCCCTTCTCCATAAT	
	*Cs*DQD/SDHb-338G→S-R	ATTATGGAGAAGGGGACTTTTGCTGTGGCCCAC	
	*Cs*DQD/SDHb-381G→T-F	GCAGGTTTCAGTGTAACATTTCCATACAAGGAA	
	*Cs*DQD/SDHb-381G→T-R	TTCCTTGTATGGAAATGTTACACTGAAACCTGC	
	*Cs*DQD/SDHb-483D→N-F	AGGGTTGTCATTTTTAACATTGATTTTGACAGA	
	*Cs*DQD/SDHb-483D→N-R	TCTGTCAAAATCAATGTTAAAAATGACAACCCT	
	*Cs*DQD/SDHb-484I→R-F	GTTGTCATTTTTGACCGTGATTTTGACAGAGCA	
	*Cs*DQD/SDHb-484I→R-	TGCTCTGTCAAAATCACGGTCAAAAAT	

	R	GACAAC
	*Cs*DQD/SDHb-485D→T-F	GTCATTTTTGACATTACTTTTGACAGAGCAAAG
	*Cs*DQD/SDHb-485D→T-R	CTTTGCTCTGTCAAAAGTAATGTCAAAAATGAC
*Cs*DQDa	*Cs*DQDa-F	GGATCCAAGAACTCAACCCTAATTTGT
	*Cs*DQDa-R	CTGCAGGATATTGTACAAGTTCACCAA
*Cs*SDHa	*Cs*SDHa-F	GGATCCATTATCGGGAAGCCTGTTGGCC
	*Cs*SDHa-R	CTGCAGTCCAGTGAACCTCTCGAATTG

Species	Designation	Accession number
Arabidopsis thaliana	AtSDH	AAF08579
Vitis vinifera	*Vv*SDH1	KU163040
	*Vv*SDH2	KU163041
	*Vv*SDH3	KU163042
	*Vv*SDH4	KU163043
Nicotiana tabacum	NtSDH1	AAS90325
	NtSDH2	AAS90324
Solanum lycopersicum	SlSDH1	AAC17991
	SlSDH2	XP_010327280
	SlSDH3	XP_004242317
Diospyros kaki	DkSDH	BAI40147
Eucalyptus grandis	EgSDH1	HO1214.1
	EgSDH2	HO4428.1

	EgSDH3	HO4427.1
	EgSDH4	BO1770.2
	EgSDH5	J00263.6
Fragaria vesca subsp. vesca	FvSDH1	XP_004302480
	FvSDH2	XP_004302479
	FvSDH3	XP_004289250
	FvSDH4	XP_004288087
Populus trichocarpa	PoptrSDH1	010G019000
	PoptrSDH2	013G029900
	PoptrSDH3	005G043400
	PoptrSDH4	014G135500
	PoptrSDH5	013G029800
Juglans regia	JrSDH	AAW65140
